# Treatment goals in ANCA-associated vasculitis: defining success in a new era

**DOI:** 10.3389/fimmu.2024.1409129

**Published:** 2024-06-13

**Authors:** Federico Alberici, Martina Tedesco, Tamara Popov, Monica Balcells-Oliver, Federica Mescia

**Affiliations:** ^1^ Nephrology Unit, ASST Spedali Civili, University of Brescia, Brescia, Italy; ^2^ CSL Vifor, Glattbrugg, Switzerland

**Keywords:** ANCA-associated vasculitis, comorbidity, extended survival, patient perspective, treatment-induced morbidity, quality of life

## Abstract

Health-related quality of life is a key contributor to overall well-being, and this is becoming an increasingly prominent factor when making therapeutic choices in the management of ANCA-associated vasculitis (AAV). Progress in available therapeutic strategies for AAV has resulted in this historically acute disease with a potentially fatal short-term outcome, becoming a relapsing-remitting chronic disorder. This new perspective on AAV means that patient survival should no longer be considered as the only major treatment target. Additional outcomes in this context that should be portrayed in order to consider a therapeutic approach as successful include patient quality of life, as well as the burden of treatment-induced morbidity. Comorbidities and impaired quality of life in patients with AAV, as with many other autoimmune diseases, may be a consequence of the disease itself as well as a result of the therapy employed. The AAV disease process may induce organ damage, including kidney failure and structural lung damage, and increase the risk of cardiovascular disease. On top of this, treatments employed to manage the disease may contribute further to the overall comorbidities burden. Furthermore, pre-existing comorbidities can increase AAV severity and may also be contraindications that limit potential therapeutic options. Quality of life is another central topic that can have a huge impact on patient wellbeing as well as adherence to treatment. Ongoing monitoring of comorbidity risk and of quality of life is thus key for successful AAV management. This process, however, may be complicated; the identification of the correct parameters on which to focus is not always straightforward and, more importantly, it is sometimes the symptoms that may appear trivial to physicians that are most detrimental to a patient’s quality of life. With these shifts in treatment capabilities and understanding of patient burden, it is necessary to adjust the treatment paradigm accordingly. Treatment success is no longer defined solely by the control of disease activity; treatment success requires holistic improvement determined through the assessment of all aspects of the disease, ranging from disease control to comorbidity risk through to the assessment of health-related quality of life. This review explores the burden of AAV itself as well as treatment-related side effects with a special focus on the tools available to measure outcomes. The management of AAV has entered a new era with a strong focus on both the management and prevention of comorbidities as well as patient-reported outcomes, both of which are now considered key factors in defining treatment success.

## Introduction

Antineutrophil cytoplasmic antibody (ANCA)-associated vasculitis (AAV) is a group of rare diseases that cause small vessel inflammation and are potentially life-threatening. (1). AAV includes granulomatosis with polyangiitis (GPA), microscopic polyangiitis (MPA), and eosinophilic granulomatosis with polyangiitis (EGPA). Together, these conditions have a combined prevalence of fewer than 5 people per 100,000 and a reported incidence of up to 1.2 per 100,000 (male-to-female ratio of between 1.07:1 and 1.48:1)^1^. (2) Although EGPA shows some overlapping features with MPA (3), its clinical heterogeneity has led to it usually being excluded from AAV interventional trials; in line with this, we will focus only on GPA and MPA in this review.

AAV pathogenesis is complex and still not completely understood. It is known that patients have a genetic predisposition for developing AAV (4), and in this context ANCA (5) and several other factors such as NETs, T-cells, and cytokines (6–8) play a central role. Most recently, accumulating *in vitro* data, pre-clinical and clinical evidence implicate the alternative pathway of the complement cascade in the development of AAV (8, 9).

AAV manifests in a myriad of ways with a broad array of signs and symptoms across the whole spectrum of severities. (1) It is possible for a single organ or several distinct organs to be affected, and the presentation is determined by the sites involved. Among all the organs potentially targeted by the disease, involvement of the lungs and the kidneys is most common, and this is associated with potentially fatal consequences. The disease manifestation and course also differ between the two main phenotypes of AAV. MPA, which is more often MPO-ANCA positive, has a high frequency of severe renal involvement at disease onset (10, 11) and the relapse risk is low. In contrast, GPA is characterized by the presence of granulomatous features, which are usually characterized by a lower degree of severity per se but a higher degree of refractivity to therapy—these manifestations may or may not be associated with vasculitic manifestations as well (12, 13)—and are usually associated with a higher relapse risk compared to MPA. Of note, there remains significant unmet treatment needs for both subgroups.

Although advances in the management of AAV have extended survival, patients still have an elevated mortality risk (14). Furthermore, even when AAV is controlled, the risk of relapse and associated organ damage remains high. It is important to note that, once developed, damage is often irreversible and accumulates throughout the course of the disease contributing significantly to a patient’s prognosis. Quantification of the extent of organ damage is therefore key since it provides insight into the likely prognosis and consequently informs treatment decisions. AAV-specific tools such as the Vasculitis Damage Index (VDI) have been developed to facilitate evaluation of the extent of AAV-induced damage and allow disease progression/control to be objectively monitored. (15) Tools are also available for measuring the burden of AAV and its treatment, eg, the Glucocorticoid Toxicity Index (GTI) (16), and AAV-Patient-Reported Outcome (AAV-PRO) (17).

This manuscript charts the evolution in AAV patient outcomes and explores the factors that should be considered in defining appropriate treatment strategies for today’s patients living with AAV. Which aspects of treatment outcome should be included in defining success and how can they be evaluated?

## AAV prognosis: from fatal dysfunction to chronic disorder

Recent studies have reported a growing population of patients with AAV. It is likely that the observed increases in AAV prevalence are at least partly due to improved disease awareness and diagnosis and faster access to effective anti-inflammatory and immunosuppressive drugs preventing potentially life-threatening organ damage. (13, 18) These once-fatal conditions are thus now considered as chronic relapsing disorders.

Early case reports of patients with AAV described widespread inflammation that for the majority of patients progressed rapidly causing death from renal or respiratory failure. Although the disease could be controlled by steroids in a few patients, the average survival was only 5 months and some patients died after only 4 weeks (19).

**Figure 1:**
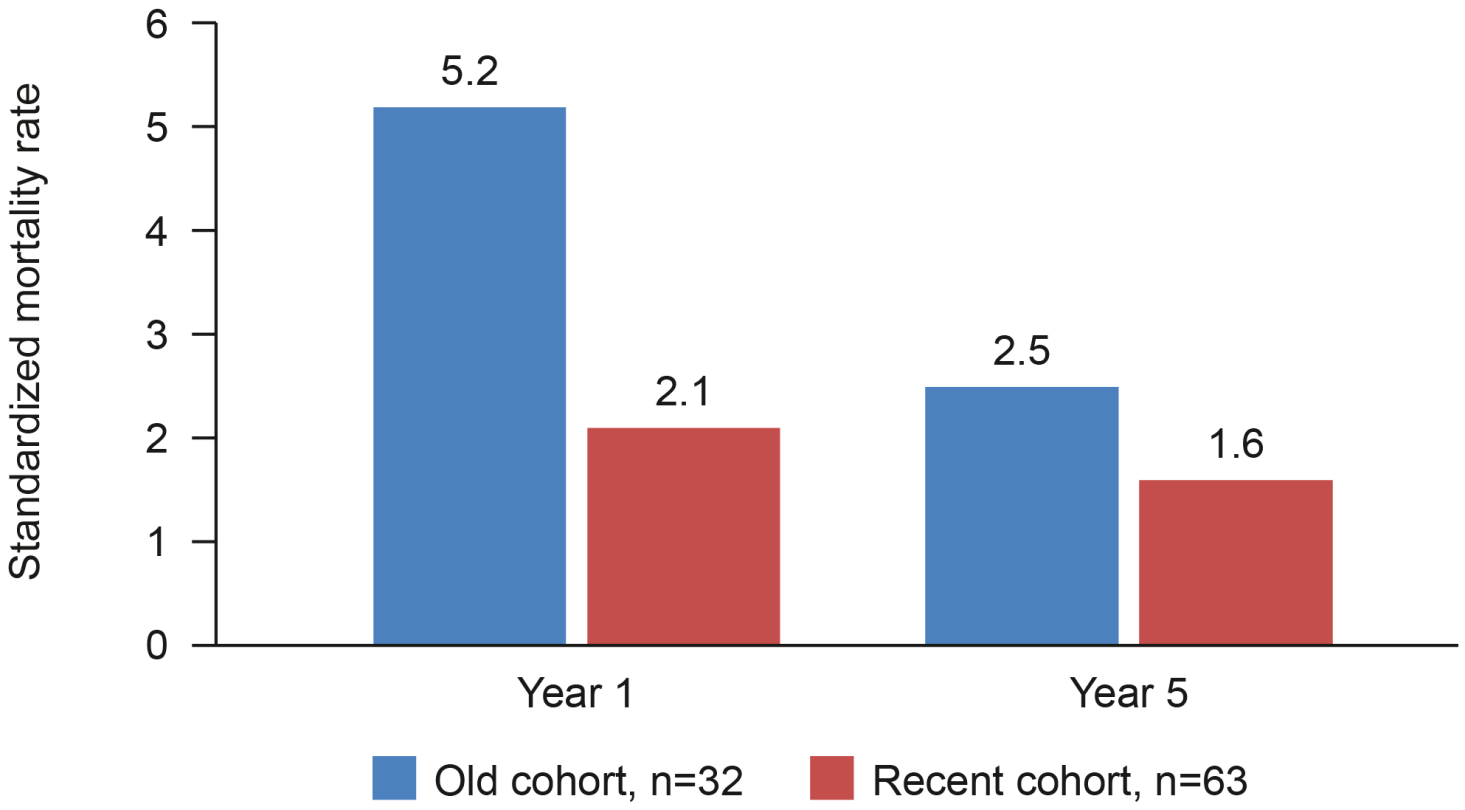
AAV mortality rate compared to general population before and after 1997. Retrospective analysis to compare survival in an historical and a more recent cohort of patients with AAV at Linköping University Hospital, Sweden (22).

The introduction of cyclophosphamide in the 1960s was a breakthrough in changing the disease course when used in combination with existing steroid treatments. Nonetheless, mortality rates were still high, being around 15–25% after 2 years, and treatment-associated toxicity was also high. (20) An analysis of 265 cases of GPA diagnosed in England, Wales and Scotland between 1975 and 1985 showed that 74 patients treated with oral cyclophosphamide +/– prednisolone had a median survival of 8.5 years. (21) With continuing refinement of treatment regimens, further survival benefits were achieved. A retrospective analysis of 95 patients with AAV reported better survival for patients diagnosed after January 1997 compared with those diagnosed earlier (**Figure 1**). Survival at 5 years was 81% for those diagnosed between 1978 and 1996 and 87% for those diagnosed between 1997 and 2005 (22).

Immunosuppressive and anti-inflammatory treatments, mainly cyclophosphamide and prednisolone, have helped control the progression of AAV and increased the mean 5-year survival rate for patients with AAV. (23, 24) However, AAV continued to be associated with an elevated mortality risk. A long-term survival analysis conducted just over a decade ago in 535 patients prospectively recruited at the time of AAV diagnosis reported a mortality risk nearly three times that of the general population (odds ratio 2.6), with a quarter of the patients dying after a median follow-up of 5 years. (25) Ongoing advancements in AAV treatment have continued to improve the prognosis, with the mean duration of survival after a diagnosis of AAV now being 17.8 years. (14, 26, 27).

The reported survival gains, although undoubtedly a success story, were typically achieved at the cost of high treatment toxicity. It was not until the advent of rituximab that there was a realistic prospect of reducing treatment-induced morbidity. (28) Notwithstanding the tremendous progress that has been made in improving the prognosis for patients with AAV, patients still experience considerable morbidity in terms of treatment complications and organ damage. (14) Furthermore, only a year after diagnosis, the mortality rate among patients with AAV is 14% higher than in the general population, and the difference increases with increasing AAV duration. (14) Of note, infections, the risk of which is increased by AAV treatments, are the leading cause of premature death.

## Recent AAV treatment advances

### Rituximab

Despite their associated toxicities, glucocorticoids and cyclophosphamide were for several decades the mainstay of AAV treatment to minimize the organ damage that rapidly accumulates in untreated disease. (29) Since there were no other options for controlling AAV, the toxicity of available immunosuppressive agents was endured to extend life. It was 2011 before a major innovation in the treatment of AAV was seen with the introduction of rituximab. (28, 30) Rituximab is a monoclonal antibody that selectively targets CD20+ B cells causing their depletion without affecting plasma cells.

AAV induction therapy with rituximab achieved similar remission rates to those with cyclophosphamide in the randomized controlled trials RAVE and RITUXVAS. (31–33) In RAVE, remission rates at 6 months were 64% for patients with new-onset or relapsing AAV who received rituximab compared with 53% for those who received cyclophosphamide (noninferiority p<0.001). Rituximab was superior to cyclophosphamide in achieving remission in the subset of patients with relapsing disease (67% vs. 42%; p=0.01). In RITUXVAS, rates of sustained remission rates at 12 months were equivalent for rituximab and cyclophosphamide in patients with severe renal AAV involvement (76% vs. 82%, p=0.68). However, since patients in the rituximab arm of the RITUXVAS trial also received 2–3 cyclophosphamide infusions, there remains some question regarding the equivalence of the two therapeutic approaches in this clinical context. Among patients, mainly with recent-onset disease, who were treated with cyclophosphamide as induction therapy, the MAINRITSAN trials demonstrated superior efficacy for rituximab over azathioprine as AAV maintenance therapy; (34–37) notably the rate of major relapse at Month 28 was 29% with azathioprine and 5% with rituximab. The RITAZAREM study evaluated the efficacy of rituximab in patients with relapsing AAV who had received rituximab induction. It demonstrated superior relapse prevention with rituximab over azathioprine. (38–40) At Month 24 relapse had occurred in 13% of patients receiving rituximab, compared with 38% in the azathioprine group (Hazard ratio 0.36, p<0.001). In the context of rituximab treatment, B-cells and ANCA may play a role as biomarker for risk of relapse (41). The possibility of guiding rituximab retreatment according to these biomarkers (“tailored approach”) was explored in the MAINRITSAN 2 trial; no difference was apparent between the so called “tailored approach” and the fixed dose schedule (as per MAINRITSAN 1), but this may have been due to the trial not being sufficiently powered. (35) Long-term follow-up of patients in the MAINRITSAN trials program has confirmed that there is a higher long-term risk of relapse among patients treated using the “tailored approach” (42). However, there is also accumulating evidence suggesting a high variability in the timing of B-cells repopulation. (43) Thus, it is possible that a “tailored approach” based on this biomarker may prove useful in specific subsets of patients (44, 45). Irrespective of this, it is clear that prolonging rituximab maintenance therapy reduces the risk of relapse. In the placebo-controlled MAINRITSAN 3 trial, 96% of the patients treated with rituximab maintenance therapy after completing MAINRITSAN 2 remained relapse-free at Month 28. (37) Adverse event data for these trials are summarized in **Table 1**.

**Table 1:** Summary of adverse event data from key rituximab and avacopan trials.

Trial	Treatments	Safety follow-up duration	Summary of safety data
Rituximab			
**RAVE** (32, 33)	Rituximab 375 mg/m^2^/week×4 plus daily placebo cyclophosphamide (n=99). On achieving remission (n=61) there was no further active treatment to Month 18 Placebo–rituximab infusions plus daily cyclophosphamide 2 mg per kilogram of body weight, adjusted for renal insufficiency (n=98). On achieving remission (n=63) azathioprine 2 mg/kg was administered to Month 18	18 months	Induction Total number of adverse events similar in both groups at Month 6 (1035 v 1016) Number of serious adverse events was similar in both groups (79 v 78) Most frequent selected adverse events^†^ with rituximab were: Hospitalization due to disease or treatment 8% (placebo 2%) Infection (≥grade 3) 7% (placebo 7%) Venous thrombotic event 6% (placebo 9%) Thrombocytopenia (≥grade 3) 3% (placebo 1%) Leukopenia (≥grade 2) 3% (placebo 10%) Infusion reaction preventing further infusions occurred in 1 patient receiving rituximab Maintenance There was no significant between-group difference in adverse event incidence at 18 months (99% v 100%) There was no significant difference between the treatment groups in the number or rate of infections of grade 3 or higher (12% v 11%) Leukopenia of grade 2 or higher was less common in rituximab group (5% v 23%, P<0.001)
**RITUXVAS** (31)	Glucocorticoids plus rituximab 375 mg/m^2^/week×4 with 2 intravenous cyclophosphamide pulses (n=33) Glucocorticoids plus intravenous cyclophosphamide for 3–6 months followed by azathioprine (n=11)	24 months	An adverse event affected 76% of patients in the rituximab group and 64% of patients in the control group The incidence of adverse events of severe intensity was similar in both groups (42% v 36%). The serious adverse event rate was 36% for both groups Infections occurred in 36% of patients in the rituximab group and 27% of patients in the control group. The incidence of serious infection was 18% in both groups. Fatal infections occurred in 3 patients in the rituximab group and in 1 patient in the control group
**MAINRITSAN1** (34)	Fixed rituximab 500 mg infusion on days 0 and 14 after randomization, and then at months 6, 12, and 18 after the first infusion (n=57) Azathioprine 2 mg/kg/day for 12 months, and then 1.5 mg/kg/day for 6 months and 1 mg/kg/day for 4 months (n=58)	28 months	25 patients in each group experienced a severe adverse event. Severe infections affected 19% of rituximab group and 14% of azathioprine group Fatal sepsis occurred in 1 patient of the azathioprine group
**MAINRITSAN1 follow-up** (36)	Azathioprine maintenance treatment by for 18 months (n=58) Rituximab 375 mg/m^2^ infusion every 6 months until month 18 (n=57)	60 months	Quality-adjusted time without symptoms and toxicity was 12.6 months longer for rituximab group (p<0.001). Serious adverse event-free survival was comparable for the two treatment groups (Hazard ratio 1.02; p=0.951) Severe infections (mainly respiratory) occurred in 28% of azathioprine group and 26% of rituximab group. There were more bronchitis events with rituximab than with azathioprine (10 v 1) There were fewer cancer events with rituximab than with azathioprine (2 v 6) Overall survival at 60 months was 100% for rituximab and 93.0% for azathioprine (p=0.045)
**MAINRITSAN2** (35)	Rituximab 500 mg infusion + further infusions with rituximab reinfusion only when CD19+B lymphocytes reappeared or ANCA titre rose markedly. (n=81) Fixed rituximab 500 mg infusion on days 0 and 14, then 6, 12 and 18 months after the first infusion. (n=81)	18 months	Adverse events affected 85% of tailored group and 91% of fixed group (p=0.33). There were 37 serious adverse events in the tailored group and 53 in the fixed group The incidence of infectious complications was 11% in the tailored group and 20% in the fixed group. The number of pneumonia events was greater in the fixed group (6 v 3) There were 3 deaths (1 due to nosocomial pneumonia) in the fixed group and 1 in the tailored group, but none was deemed to be related to treatment with rituximab
**MAINRITSAN3** (37)	Rituximab every 6 months for 18 months (4 infusions) (n=50) Placebo infusion every 6 months for 18 months (4 infusions) (n=47)	18 months	Adverse events occurred in 92% of the rituximab group and 94% of the placebo group (p = 0.68). At least 1 serious adverse event developed in 12 patients (24%) in the rituximab group versus 14 patients (30%) in the placebo group (p = 0.65) The incidence of serious infections was 12% with rituximab and 9% with placebo. Treatment was discontinued due to an adverse event for 4 (8%) rituximab patients and 1 (2%) placebo patient. No deaths occurred in either group
**RITAZAREM** (39); (46)	Re-induction with rituximab 375 mg/m^2^/week x4 and glucocorticoids (N=187) followed by Rituximab maintenance (n=85) OR Azathioprine maintenance (n=85)	48 months	Induction phase At Month 4, severe adverse events had occurred in 27 patients, including 13 severe infections. Five of the 13 severe infections occurred within 4 weeks of the first induction dose of rituximab. There were 86 non-severe infections Four patients died due to pneumonia (n=2), cerebrovascular accident (n=1), and active vasculitis (n=1) Maintenance phase The incidence of non-serious adverse events was similar in both groups (49% v 51%). Serious adverse events affected 19 (22%) patients in the rituximab group and 31 (36%) in the azathioprine group There were 19 serious infections in the rituximab group and 24 in the azathioprine group. A new malignancy developed in 5 patients in the rituximab group and 6 in the azathioprine group. There were 3 deaths (including 1 infection and 1 malignancy) in the rituximab group and 1 (malignancy) in the azathioprine group
Avacopan			
**ADVOCATE** (47)	30 mg of avacopan twice daily orally for 52 weeks plus prednisone-matching placebo (n=166) versus tapering 20 weeks oral regimen of prednisone plus avacopan-matching placebo (n=165) Along with cyclophosphamide followed by azathioprine, or rituximab	52 weeks	The incidence of adverse events was similar in both groups (99% v 98%) The incidence of serious adverse events was similar in both groups (42% v 45%) There were two deaths in the avacopan group and four deaths in the prednisone group There were 9 events of serious adverse liver function abnormalities in the avacopan group (5.4%) and 6 events (3.7%) in the prednisone group There were 22 serious infections in the avacopan group (13.3%) and 25 (15.2%) in the prednisone group Life-threatening adverse events were less common with avacopan (4.8% v 8.5%)
**Pooled analysis** (48)	Avacopan 10 mg twice daily (n = 13) or 30 mg twice daily (n = 226) versus a prednisone taper (n = 200) along with cyclophosphamide followed by azathioprine, or rituximab	12 or 52 weeks	Rate of exposure-adjusted total adverse events/100 patient years was 1099.8 in the avacopan group and 1251.7 in the prednisone group Serious adverse event rate was lower with avacopan (70.7 v 91.5/100 patient years) Infection rate was lower with avacopan (142.2 v 166.6/100 patient years)

^†^Included deaths (from all causes), malignant conditions, grade 2 or higher leukopenia or thrombocytopenia, grade 3 or higher infections, drug-induced cystitis, venous thromboembolic events, stroke, hospitalizations, and infusion reactions that contraindicated further infusions.

Rituximab has indeed revolutionized the management of AAV and can achieve full drug-free remission in some patients, however, the treatment is not curative in the majority of cases; relapse rates are very high and even after successful rituximab maintenance treatment a return of B-cells and a rise in ANCA frequently occurs (41).

In the context of a drug that has revolutionized the therapeutic scenario in the field of AAV, the tolerability of rituximab is generally good, but side effects are still common (30). Infusion reactions are relatively frequent with rituximab administration, but this is usually easily managed with pre-medication or treatment with anti-histamine and glucocorticoids. (49) The greatest risk of rituximab treatment is an increased risk of infection and the development of rituximab-associated hypogammaglobulinemia, which can further increase susceptibility to infection. (50–52) In the RAVE trial, the incidence of infectious complications reported was similar for rituximab and cyclophosphamide (32), although this may have been due to the concomitant use of glucocorticoids; further evaluation of the link between rituximab and infection risk is required. Importantly, while vaccination is one of the main tools to mitigate infection risk in patients on immunosuppression, B cell depletion also impairs humoral response to vaccines, which should ideally be administered after B cell repopulation to maximize the chances of response (53, 54).

Furthermore, rituximab treatment carries the risk of late-onset neutropenia, which can develop after 6–8 months, although patients usually recover without treatment (40).

### Avacopan

In 2021 avacopan was approved for the treatment of AAV. Avacopan is a selective C5a receptor that blocks the effects of C5a, a key product resulting from activation of the alternative complement pathway that has been strongly implicated in the pathogenesis of AAV (55).

Avacopan was non-inferior to prednisone at inducing clinical remission in patients with severe GPA or MPA on a background of rituximab or cyclophosphamide after 6 months in the ADVOCATE trial (72.3% and 70.1%, respectively; p<0.0001) (47).

In addition, the relapse rate in the avacopan group was half that in the prednisone group (10.1% vs 21.0%) and rates of sustained remission at one year were significantly higher with avacopan (66% vs 55%; p=0.007). Treatment with avacopan reduced the need for glucocorticoids and this was reflected in a lower glucocorticoid toxicity score at Week 26 (11.2 in the avacopan group and 23.4 in the prednisone group). An unanticipated benefit of avacopan was the impact on kidney function. A more rapid reduction of proteinuria was observed in the avacopan treated arm, together with an estimated glomerular filtration rate (eGFR) improvement more significant with avacopan than with prednisone (7.3 vs 4.0 mL/min/1.73 m^2^; p=0.0259). Adverse event data are summarized in **Table 1**; a post-approval safety study (AVACOSTAR, NCT05897684) is ongoing to evaluate long-term safety.

A pooled analysis of data from the three key controlled clinical trials of avacopan versus prednisone in AAV did not reveal any tolerability concerns for avacopan (48). The incidences of serious adverse events and infections were lower in the avacopan group than in the prednisone group.

## Impact of AAV treatment on quality of life

With the high relapse rate in AAV, long-term treatment is often required to manage the risk of flare, and the resultant treatment-related toxicities give rise to a high level of morbidity. (26, 56, 57) Historically, the focus of AAV treatment was necessarily skewed towards limiting organ damage in order to prevent death; however, it has since been realized that treatment-related side effects may be as deleterious as the vasculitis itself. Indeed, the occurrence of treatment-related causes of death are significant at every stage of AAV management. (25) Research efforts have now been focused for some time on finding ways to limit treatment-related toxicity and reduction in quality of life and studies will need to focus specifically on the long-term outcomes of such approaches in terms of damage accrual throughout the time. (58) Nonetheless, despite reports showing that even with well-controlled disease activity, people with AAV have lower quality of life than the general population, the impact on quality of life is still not routinely and systematically addressed in treatment plans for AAV. (59)

AAV treatments commonly give rise to a range of adverse effects that, although minor in a medical context, may be significant from the patient’s perspective. Insomnia, nausea, dizziness, and fatigue can limit a patient’s ability to participate in everyday activities thereby having a significant detrimental impact on their quality of life. (57, 60–62) Indeed, many patients report that they find these side effects more troublesome than the disease itself. (17, 63) Similarly, the development of apparently trivial cold-like symptoms, such as rhinitis, mouth ulcers, sore throat, cough, have the potential to significantly impact on quality of life as well by interfering with sleep to cause fatigue (64). Fatigue is reported by patients with AAV as having the most detrimental effect on quality of life and it results in more work disability claims than AAV itself. (65, 66) It should be remembered that multi-morbidity is usual among patients with AAV, and so these effects are rarely experienced in isolation, thereby amplifying the detrimental impact on quality of life. (58) A recent analysis of 543 patients with AAV diagnosed between 1997 and 2017, each matched with up to 5 general population controls, confirmed that multimorbidity was significantly more likely with AAV, especially during the first 2 years after diagnosis. (67) After 1 year, multimorbidity affected 23.0% of AAV patients compared with 9.3% of controls (p<0.0001).

Serious comorbidities as a consequence of prescribed medications are also a real risk in AAV. In fact, an analysis of 6 randomized trials conducted by the European Vasculitis Study Group (EUVAS) showed that much of the long-term damage, eg, cardiovascular disease, malignancy, osteoporosis, occurring in newly diagnosed patients with AAV was potentially treatment-related. (68) Similarly, it has been reported that the main cause of death within the first year of an AAV diagnosis is therapy–related adverse events (59%). (69) In an evaluation of four European AAV trials, the adverse event most commonly resulting in death was infection, being cited for half of all mortalities within the first 12 months. Cardio/cerebrovascular disease was the second most common fatal adverse event accounting for 13% of deaths. A multivariate analysis found renal impairment and infection, but not age or vasculitis type, to be independently predictive of early mortality (69).

The treatment-induced morbidities commonly encountered by patients with AAV include increased infection risk, which is exacerbated by treatment-induced hypogammaglobulinemia as well as cardiovascular disease, increased risk of malignancy, osteoporosis, cataracts and glaucoma, increased risk of developing a neuropsychiatric disorder such as depression, insomnia, and akathisia. These treatment-induced comorbidities often require hospitalization or additional medication, further compromising health-related quality of life through the requirement for additional clinic visits and hospital admissions. (70, 71) For example, the incidence of osteoporosis was recently found to be 8-fold higher in AAV than in the general population and the risk of hospitalization because of hip fracture was two-fold higher among patients with AAV (67).

Each adverse effect and comorbidity contributes to the morbidity burden on the patient and an individual patient may have several to contend with concomitantly, in addition to the symptoms associated with the disease itself. (59, 65) Although the symptoms of aggressive vasculitis and its complications are key factors reducing health-related quality of life in AAV, it is clearly apparent that AAV treatment can have significant emotional, physical, and social impacts on the patient, with severely detrimental effects on quality of life (65, 72, 73).

The high contribution of AAV treatments to the development of chronic morbidities with associated detrimental impact on a patient’s quality of life highlights the need for current AAV management strategies to be re-evaluated.

## Current treatment of AAV

The evidence base supporting different treatment strategies is continually growing and consequently treatment recommendations evolve accordingly. Advances in medical technology have enabled the development of biological therapies that can be used to control the aberrant immune responses observed in AAV, e.g., with rituximab. In addition, research during the last decade into the pathogenesis of AAV has greatly increased our understanding of the underlying causes of AAV. Not least identification of the involvement of the alternative complement pathway, which has provided a novel target for pharmacological intervention, eg, with avacopan. It is thus now possible to use targeted therapies to alter the disease course of AAV rather than just manage the symptoms. The specificity of such agents also reduces the potential for side effects. Furthermore, availability of additional options for effectively controlling the AAV disease process will help reduce the reliance on more toxic treatments, such as glucocorticoids. Optimal use of these agents thus has the potential to improve outcomes for patients with AAV.

### Induction

EULAR treatment recommendations have recently been substantially revised to reflect the current evidence base for AAV management. One of the more significant amendments in the latest EULAR guidelines is the inclusion of the recommendation to use avacopan in combination with rituximab or cyclophosphamide as a glucocorticoid-sparing regimen for the induction of remission in GPA or MPA. (74) Indeed the current EULAR guidelines highlight the reduction of glucocorticoid exposure to improve patient wellbeing as a key goal in the management of AAV, and several of the latest recommendations are focused on achieving this. A stepwise reduction in glucocorticoid dose is recommended from a starting dose of 50–75 mg/day to prednisolone 5 mg equivalent per day by 4–5 months. Rituximab is recommended over cyclophosphamide to enable achievement of remission using a lower glucocorticoid dose and with a lower risk of complications. Similarly, the use of avacopan in combination with rituximab is recommended for induction of remission in GPA or MPA to substantially reduce glucocorticoid exposure. Unfortunately, the cost of innovative drugs may be seen as a barrier and limit their full implementation in clinical practice, although available study data seem to suggest a favorable cost-effectiveness profile for an induction regimen combining avacopan and rituximab (75, 76).

In addition, research has shown that the dose of glucocorticoids used can be reduced without compromising efficacy and that this significantly reduced the occurrence of adverse effects. (77, 78) Most recently, in the LOVAS trial AAV remission rates at six months were similar for standard-dose and reduced-dose glucocorticoids (69% and 71%, respectively), but the rate of serious infections was significantly higher among patients who received the standard dose (20% vs. 7%, p=0.04). (79). Despite compelling data from several studies, it appears from a recent evaluation of current treatment practices that glucocorticoid dose is not being reduced for the majority of patients in clinical practice. (80) A retrospective clinical audit of healthcare records for AAV patients managed by 493 consultants across Europe found that the use of high-dose glucocorticoids was common in all the countries studied and frequently continued for 36 months or more. Even more concerning is the reality that, despite enduring treatment-induced morbidity, many patients with AAV continue to experience frequent relapses and for some patients AAV remission has not been achieved (16, 80, 81).

### Maintenance

The latest EULAR treatment recommendations for maintenance treatment favor rituximab over azathioprine due to its superior success record in relapse prevention. Rituximab may be especially preferable in patients with relapsing forms of AAV (74). The MAINRITSAN studies showed relapse prevention to be better with rituximab than with azathioprine up to a follow-up of 60 months and that prolonging rituximab treatment for an additional 18 months was effective in sustaining remission (36, 37). Similar superiority for rituximab among patients with relapsing AAV was demonstrated in the RITAZAREM trial. (39) However, since both the patient profile and the rituximab treatment schedule differed between the MAINRITSAN and RITAZAREM trials, there remain unanswered questions regarding the ideal maintenance regimen. Several randomized controlled trials are underway to try and determine an optimal rituximab regimen, e.g., ENDURRANCE1 (NCT03942887), MAINTANCAVAS (NCT02749292). The MAINRITSAN2 trial found no benefits in terms of efficacy or adverse event rates with flexible rituximab dosing driven by B-cell repopulation or ANCA rise over the fixed dosing used in MAINRITSAN1. (35) AAV experts tend to agree that a fixed-dose approach is safer in terms of relapse prevention although a subset of patients, especially those with MPO-ANCA positive MPA with kidney involvement and at the first flare of the disease, may experience high variability in the kinetics of B-cells repopulation with patients experiencing longer B-cells depletion being less likely to require a structured fixed-dose maintenance approach (43).

### Renal impairment

**Figure 2:**
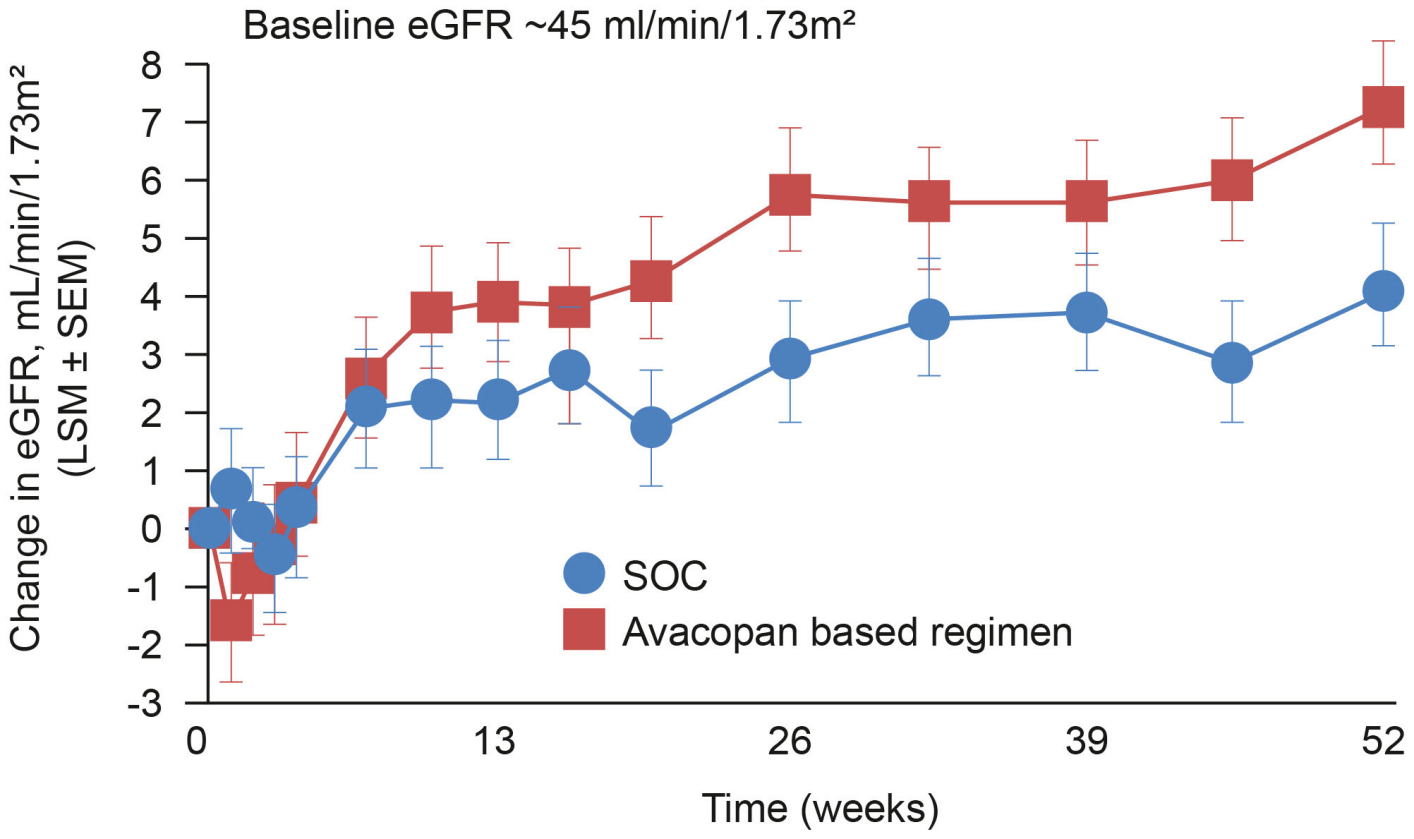
Change in eGFR among patients in the ADVOCATE trial with renal disease at baseline. Patients with AAV were randomized to receive either a tapering prednisone schedule for 20 weeks (n=164) or avacopan for 52 weeks (n=166) in combination with cyclophosphamide (followed by azathioprine) or rituximab. (47). eGFR, estimated glomerular filtration rate; SOC, standard of care.

Renal involvement is very common in GPA and MPA and typically shows rapid progression of glomerulonephritis with resultant renal failure. (82) The potential for improvements in renal function with avacopan (**Figure 2**), especially amongst those patients with severe renal involvement, (83) is therefore of particular interest considering the significant detrimental impact of chronic kidney disease on a patient’s life expectancy and quality of life, as well as the burden on healthcare resources. Interestingly, the significant improvements in kidney function observed in patients receiving avacopan during the ADVOCATE trial were preceded by a transient initial decline in eGFR compared to the placebo arm (**Figure 2**). The reason for that is unclear and will require further investigations; however, especially in the light of the rapidity of the effect and the transiency of the phenomena, the most likely explanation is a functional reason. On this perspective, is to be noted the well-known effect of glucocorticoids in increasing GFR (84, 85) leading to the possible speculation that the early drop in eGFR of the avacopan group may in fact be an unmasking of a glucocorticoids induced hyperfiltration in the context of their quick tapering.

Clinical trial data for avacopan support an early improvement of kidney inflammation as measured by reductions in proteinuria and MCP-1. (86) In contrast, biopsies from patients with complement factor 3 (C3) glomerulopathy support a role for avacopan in reducing chronicity features over time. (87) It will be interesting to explore whether avacopan can impact not only the rate of improvement of eGFR after the vasculitis flare but also the subsequent slope of eGFR reduction over time after remission has been achieved and kidney function stabilized.

The effects of avacopan on kidney function are not completely understood and an evaluation of long-term effects and benefits is required.

### Treatment moving forward

Despite tremendous advances, there remain several unmet needs critical to the successful management of AAV. Induction treatments do not always effectively control disease activity and patients continue to receive immunosuppressive treatment without achieving remission. This allows irreversible tissue damage to accumulate whilst increasing the risk of serious infectious complications. Even when remission is achieved, the risk of relapse is high, contributing to damage accrual, which in turn can lead to serious complications and impact survival. In addition, many patients still progress to kidney failure and other comorbidities, such as cardiovascular disease, adding to the patient morbidity burden and increasing mortality risk.

Unanswered questions regarding the required duration of AAV treatment may also result in some patients being exposed to unnecessary treatment and the associated risk of side effects. Despite the MAINRITSAN2 trial not finding an advantage to individually tailor rituximab dosing, identification of specific biomarkers to guide remission-maintenance strategies may help limit patient exposure to unnecessary treatments (88).

Real-world data assessing treatment strategies in clinical practice are needed to describe how the available treatments are being prescribed in clinical practice, determine the extent to which treatment recommendations are being adopted, and evaluate how successful they are in non-clinical trial settings.

## Evaluating treatment success in AAV

The scope of treatment options available for AAV is expanding, with avacopan now included in treatment guidelines and other pipeline products showing promise. (89) This is positive news for patients with AAV, but it is also important to evaluate whether the availability of more effective treatments is translating to better patient outcomes. To evaluate this, we must consider what successful treatment means. Is it purely the prevention of life-limiting AAV activity, or should we be taking a broader view?

Controlling AAV progression and maintaining remission are indeed important objectives in the treatment of AAV, but the right balance with treatment-induced morbidity has to be found. (1, 67) AAV is nowadays considered to be a chronic, relapsing-remitting disorder rather than a fatal condition, and so the parameters for defining treatment success need to be adjusted accordingly. The value of controlling the progression of AAV is somewhat reduced if the patient is too fatigued or has too great a comorbidity burden impacting on the everyday life.

Maintenance of remission is obviously a key goal of AAV treatment, but it is increasingly important that this clinical success is balanced with patient quality of life. (58) As we have seen, multi-morbidity is a key driver of poor quality of life in patients and seemingly trivial adverse effects can often feel more detrimental to the patients than the disease itself. Consequently, a patient may have good control of their AAV but still feel dissatisfied with the treatment outcome. It follows that evaluations of treatment success should incorporate a patient perspective and consideration of all symptoms rather than just those of greatest medical concern.

To facilitate incorporation of the patient’s perspective into evaluations of treatment success in AAV, a disease specific questionnaire, the AAV-PRO, has been validated to collect patient opinions on the impact of AAV and its treatment on symptoms, side effects, physical function and social activity and emotional wellbeing. (17, 90) Such evaluation could help increase the focus on managing the symptoms that are most troublesome to the patient. For example, ear nose and throat involvement in AAV is often side-lined as a less significant aspect of the disease yet the associated symptoms can considerably impact a patient’s quality of life. (64).

Quality of life is a complex concept as it can differ from one person to another according to their expectations and desires; moreover at certain stages of the patients journey it may not be straightforward to identify which factors are impacting more significantly on patients quality of life (disease activity vs disease induced damage vs medication related side effects). A given symptom or side effect can be viewed completely differently by different patients; one patient may accept it as an inconvenience whereas it could be viewed as devastating to another for whom it precludes participation in a much-loved activity or enforces undesirable lifestyle changes. Evaluation of health-related quality of life is thus useful to gauge how a disease and its treatment are impacting an individual patient’s life. Its value is reflected in the range of tools available for measuring health related quality of life, eg, 36-Item Short-Form SF-36, EuroQoL 5 Domain (EQ-5D) tool, CDC-HRQOL. Of note, quality of life assessments has only recently started to be included in AAV studies. However, the information they have provided is not always straightforward to interpret. For example, although deemed to be a superior and better tolerated treatment, improvements in quality of life among patients receiving rituximab were no greater than for those receiving cyclophosphamide. (32) It is likely that the co-administration of corticosteroids may have acted as a major confounder in this perspective. The first study in the AAV setting to show statistically significant improvements in health-related quality of life between the treatment arms was ADVOCATE. A *post-hoc* analysis of health-related quality of life changes in the ADVOCATE trial showed that patients receiving avacopan reported significant and clinically meaningful improvements at 52 weeks compared to baseline in EQ-5D and SF-6D scores. (91). Differences in reported health-related quality of life between patients treated with avacopan versus prednisone are at least partly explained by the differing doses of glucocorticoids, reflected in the negative mean change in general health perceptions versus improvement with avacopan. However, the substantial beneficial effects on health-related quality of life at week 52 with avacopan treatment compared with prednisone are likely to be explained by additional factors other than glucocorticoid use, that are yet to be elucidated. Ensuring that patients feel positive about their treatment is a key aspect of recovery and wellbeing, so addressing the disease and treatment issues that most concern a patient will help to improve outcomes overall.

Patients with AAV are at heightened risk of developing comorbidities, arising either as a complication of AAV-induced damage or due to adverse treatment effects. These risks remain even when AAV is in remission since considerable irreversible tissue damage arises early in the disease course, which can result in the development of comorbidities at a later stage. (68) Consequently, it is important to determine the extent of AAV-induced damage, eg, using the Vasculitis Damage Index (VDI) (92) and to evaluate the risk of this damage causing future comorbidities. This requires an holistic approach in which disease status, in the context of other risk factors a patients may have, is evaluated by a multi-disciplinary team on an individual patient basis to reduce the risk of future morbidities, eg, maintaining/improving kidney function, reducing fracture risk, maintaining cardiovascular health. A patient may already have comorbidities at the time of diagnosis, and these can exacerbate the impact of AAV (93).

An appraisal of comorbidity risk is also important when evaluating the additional potential morbidity risks of a treatment for a given patient when making decisions on treatment strategy. In this way, the choice of AAV treatment could be tailored on an individual basis according to their susceptibilities to certain comorbidities. For example, models have been developed to estimate whether a patient is at high risk of developing an infection to help inform decisions regarding the potential benefit of ongoing rituximab treatment. (94) Similarly, it may prove to be beneficial to choose options with a well-defined impact on kidney function in AAV patients with renal involvement. By minimizing risk, future outcomes can be improved by avoiding more serious manifestations and the need for hospitalization.

Evaluating success and optimizing outcomes in the management of AAV thus requires routine monitoring using a combination of assessments to evaluate the extent of AAV damage, susceptibility to comorbidities, and the impact of AAV symptoms and adverse events on the patient as well as AAV disease activity, and treatment strategy modified accordingly. Key validated tools (95) available for measuring patient outcomes are outlined in **Table 2**. A recent evaluation of the performance of tools for assessing outcome found the instruments with the best performance in AAV were the Birmingham Vasculitis Activity Score (BVAS) for disease activity, the VDI for tissue damage, and the AAV-PRO for health-related quality of life (96).

**Table 2:** Key tools available for evaluating outcomes in AAV.

Tool	Purpose	Completed by	Further details
**BVAS** (97)	Measure the level of disease activity, by identifying all the possible organ clinical manifestations	Physician	Score is divided into 9 organ-based systems. Includes both persistent and new or worsening signs and symptoms deemed to be due to vasculitis.
**VDI** (92)	Distinguish vasculitis-induced chronic damage from active inflammation or persistent disease	Physician	Comprises 64-item checklist in 11 categories. Does not differentiate between disease- and treatment-induced damage.
**AAV-PRO** (17 **;** 98)	Provide AAV-specific evaluation of patient-reported quality of life and patient perception of disease and treatment effects	Patient	Comprises 35-item questionnaire relating to patient-perceived impact of AAV and its treatment on various aspects of life Validated tool to collect patient opinions on the impact on symptoms, side effects, physical function and social activity and emotional wellbeing.
**SF-36** (99)	Generic tool for evaluating health-related quality of life	Patient and physician	Comprises 36 questions that cover eight domains of health
**GTI** (100) (101)	Measure change in glucocorticoid morbidity over time	Physician	Include 31 symptoms of toxicity
**HAQ** (102)	Patient-reported evaluation of functional status	Patient	Comprises 20 items in eight domains related to measuring difficulty in performing daily activities
**EQ-5D** (103)	Generic tool for evaluating health-related quality of life	Patient and physician	Includes one question for each of five dimensions (mobility, self-care, usual activities, pain/discomfort, and anxiety/depression) and a visual analogue scale (0–100) for patient to rate their perceived health status.

AAV-pro, AAV patient-reported outcomes; BVAS, Birmingham Vasculitis Activity Score; EQ-5D, EuroQol- 5 Dimension Questionnaire; GTI, Glucocorticoid Toxicity Index; HAQ, Health Assessment Questionnaire; SF-36, 36-Item Short-Form Health Survey; VDI, Vasculitis Damage Index.

## Concluding remarks

Medical advances have made it possible for patients to routinely survive a diagnosis of AAV.

This has shifted the parameters for defining successful AAV treatment from acute survival to long-term organ damage control as well as preservation of quality of life.

Health-related quality of life is thus of increasing importance and the goal of AAV treatment is to provide disease control while also maintaining patient health-related quality of life. This includes the management of AAV symptoms that are not life-threatening yet are particularly troublesome to the patient, such as rhinitis and fatigue. A key aspect of achieving this is obtaining the patient’s perspective of treatment success and using an AAV-specific tool for evaluating patient-reported outcomes, such as the AAV-PRO. Taking action to enhance patient quality of life will serve to improve overall patient outcomes of AAV treatment. Treatment decisions should thus consider both clinical need and patient satisfaction.

The increasing scope of effective targeted therapies provides more options to enable the tailoring of treatment to effectively balance relief from AAV symptoms with an acceptable side effect profile that meets patient needs and provides good long-term quality of life. The addition of targeted AAV treatments with fewer adverse effects to the armamentarium provides a realistic opportunity to considerably reduce the side-effect burden of AAV treatment. Optimal use of these agents remains to be defined, but the fine-tuning of therapy regimes and treatment duration could further reduce the risk of side effects.

The elevated risk of comorbidities (both disease- and treatment-induced) and high multimorbidity burden, apparent even during early stages of the disease course, emphasize the importance of multidisciplinary, holistic care for patients with AAV to assess and manage increased comorbidity risk. Current treatment plans should be designed with future implications in mind to optimize patient outcomes long-term and not just control observed disease activity.

Although there remain several challenges to the successful management of AAV, not least improving the measures of disease activity and risk of relapse, there is opportunity now to tailor treatments according to comorbidity risk and address the symptoms that as most troublesome from the patient’s perspective. Designing a treatment plan that provides patient satisfaction as well as minimizing the impact of AAV damage both currently and in the future is key to achieving success in the management of AAV.

## Author contributions

FA: Conceptualization, Writing – original draft, Writing – review & editing. MT: Writing – original draft, Writing – review & editing. TP: Writing – original draft, Writing – review & editing. MB: Writing – original draft, Writing – review & editing. FM: Writing – original draft, Writing – review & editing.
